# Effect of 2″-*O*-Rhamnosyl Icariside II, Baohuoside I and Baohuoside II in Herba Epimedii on Cytotoxicity Indices in HL-7702 and HepG2 Cells

**DOI:** 10.3390/molecules24071263

**Published:** 2019-04-01

**Authors:** Lin Zhang, Ting Wang, Bao-Sheng Zhao, Jing-Xuan Zhang, Song Yang, Chun-Lan Fan, Pin Li

**Affiliations:** Beijing Research Institute of Chinese Medicine, Beijing University of Chinese Medicine, Chaoyang District, Beijing 10029, China; zlbucm@163.com (L.Z.); zhaobs1973@163.com (B.-S.Z.); zh_xyj@126.com (J.-X.Z.); fengyasong_100@163.com (S.Y.); fanchunlan77@163.com (C.-L.F.); 20170941210@bucm.edu.cn (P.L.)

**Keywords:** cytotoxicity, 2″-*O*-rhamnosyl icariside II, baohuoside I, baohuoside II

## Abstract

Herba Epimedii, a commonly used Chinese medicine, has attracted much attention recently because of its potential hepatotoxic effects. 2″-*O*-Rhamnosyl icariside II, baohuoside I and baohuoside II are the main components of Herba Epimedii, and previous research indicates that these three compounds are related to the hepatotoxicity of Herba Epimedii. To test this idea, in this study, HL-7702 and HepG2 cells were chosen as the in vitro models and the influences of these three compounds on a series of cytotoxicity indices, including ALT, AST, LDH, SOD, GSH, MDA, ROS and MMP, were determined. The results showed that at certain concentrations, the three compounds had different effects on the indices. Among them, baohuoside I at high concentration (32 μg/mL) displayed more significant cytotoxicity than the other two compounds; therefore, it was inferred to be more closely correlated with the liver injury induced by Herba Epimedii combined with the previous study, and its toxic mechanisms may be involved in increasing oxidative stress and inducing apoptosis. The findings of this study may provide evidence of the toxic composition of Herba Epimedii to preliminarily discuss the toxic mechanisms and provide improved guidance for its clinical safety.

## 1. Introduction

Drug-induced liver injury (DILI) is an unresolved clinical problem and is the most frequent cause of post-marketing withdrawal of new medications [1]. The incidence of DILI, which ranges from one in ten thousand to ten in one million [2], is low, but DILI has a serious impact well beyond the number of actual cases that occur annually. DILI can affect both parenchymal and nonparenchymal cells of the liver, leading to a wide variety of pathological changes, mainly including acute and chronic hepatocellular steatosis, fibrosis, cirrhosis, cholestasis and hepatitis [3,4]. The predominant clinical manifestations of DILI are acute hepatitis, cholestasis, and a mixed pattern [5]. However, there are still serious limitations in current knowledge regarding the clinical diagnosis of DILI and the hepatotoxic prediction of drugs due to various confounding factors. As a result, many potential hepatotoxic drugs fail to be identified in time during clinical trials or application, and their DILI has been ignored or underestimated and has damaged public health and drug development. To better evaluate a drug’s potential hepatotoxicity and more effectively reduce the occurrence of DILI, it is imperative to analyze the reasons and explore the mechanisms of DILI.

Traditional Chinese medicines (TCMs) have been widely used throughout the world. Outside of the ethnic Chinese population, TCMs or herbal medicines are becoming more accepted in Western society based on their perceived effectiveness in the treatment and prevention of disease or the characteristics of “natural safety” [6,7]. However, TCMs may elicit adverse reactions, as all drugs do. Among the TCM-induced toxic effects, DILI has become increasingly prominent because of a causal relationship between certain TCM ingredients and specific liver toxicity [8,9]. Herba Epimedii (family Berberidaceae, Ying Yang Huo in Chinese) is a famous TCM and has the effect of warming and tonifying “Kidney Yang”; this medicine is widely used for treating sexual dysfunction and osteoporosis [10,11]. There are over 50 species in the *Epimedium* genus in different areas of China [12,13], five of which are used as medicinal materials of Herba Epimedii according to the Chinese Pharmacopoeia Commission’s suggestion [14]. In recent years, many studies have reported the potential for DILI in Herba Epimedii and its preparations [15,16,17,18], and safety issues associated with Herba Epimedii have drawn increased attention. In our previous study [19], we preliminarily demonstrated the liver damage induced by Herba Epimedii though an experiment of long-term toxicity in rats. Then, we identified the components and analyzed the toxic basis of Herba Epimedii extract by HPLC-MS/MS and filtered out certain toxic compounds in Herba Epimedii according to an experiment examining cytotoxicity in HL-7702 and HepG2 cells. Besides, we have found the Herba Epimedii extract had some effects on enzymatic activity, mitochondria oxidative stress and functional alteration of liver cells in vitro. In the next step, we plan to perform a systematic study of cytotoxicity for major suspects of these toxic compounds.

**Figure 1:**
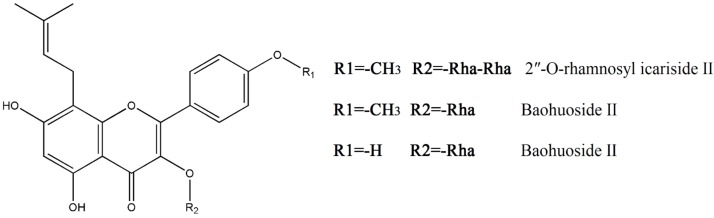
Chemical structures of 2″-*O*-rhamnosyl icariside II, baohuoside I and baohuoside II. (Rha = rhamnose).

Among these screened toxic compounds, three monomers, 2″-*O*-rhamnosyl icariside II, baohuoside I and baohuoside II, caught our attention. All these three compounds present the same skeleton of an 8-isopentene group flavonoid and only differ in certain substituting groups (Figure 1). Baohuoside I is the main metabolite of the active ingredients in Herba Epimedii, which include icariin, epidemin A, epidemin B and epidemin C [20,21,22,23]. The structure of baohuoside II results from loss of a methyl on R1 from baohuoside I. 2″-*O*-Rhamnosyl icariside II is the intermediate product in the metabolism of epidemin C into baohuoside I [23].

In view of their similar structures and close connections in chemistry and biology between 2″-*O*-rhamnosyl icariside II, baohuoside I and baohuoside II, in this study, we adopt the normal human liver cell line (HL-7702) and human hepatocellular carcinoma cell line (HepG2) as the in vitro models of the liver in order to ensure the research reliability and study their toxicity. By investigating a range of toxic indicators, we further explore the relationship between the key ingredients and toxic effects and primarily discuss the toxicity mechanism of Herba Epimedii. These results will provide guidance in developing more effective strategies to ensure the continuing safe use of Herba Epimedii.

## 2. Results

### 2.1. Effects of the Drugs on the Release of ALT, AST and LDH

**Figure 2:**
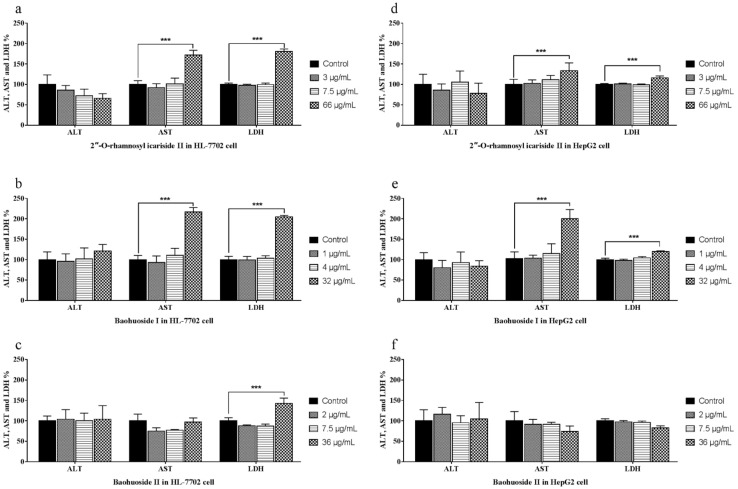
Effects of 2″-*O*-rhamnosyl icariside II, baohuoside I and baohuoside II on the ALT, AST and LDH activities in HL-7702 and HepG2 cells at 24 h. Notes: The levels of ALT were not changed after treatment with three drugs at each concentration in HL-7702 (**a**–**c**) and HepG2 (**d**–**f**) cells. The levels of AST significantly increased after treatment with 2″-*O*-rhamnosyl icariside II and baohuoside I at high concentration (*p* < 0.005) in HL-7702 (**a**,**b**) and HepG2 (**d**,**e**) cells. The levels of LDH significantly increased after treatment with three drugs at high concentration (*p* < 0.005) in HL-7702 (**a**–**c**), whereas in HepG2 cells, only 2″-*O*-rhamnosyl icariside II and baohuoside I at high concentrations caused significant increases (*p* < 0.005) (**d**,**e**). Each value is the mean ± SD of five experiments. * *p* < 0.05, ** *p* < 0.01, and *** *p* < 0.005 compared with the control group.

The activities of ALT, AST and LDH in the medium were measured to investigate the effects of 2″-*O*-rhamnosyl icariside II, baohuoside I and baohuoside II. The ALT, AST and LDH activity measurement results are shown in Figure 2, where the three drugs did not lead to an increased release of ALT in HL-7702 (Figure 2a–c) and HepG2 (Figure 2d–f) cells. 2″-*O*-Rhamnosyl icariside II at 66 μg/mL and baohuoside I at 32 μg/mL caused a significant increase (*p* < 0.005) only in AST and LDH release in both types of cells (Figure 2a,b,d,e). Baohuoside II at 36 μg/mL caused a significant increase (*p* < 0.005) in the release of LDH in HL-7702 cell (Figure 2c) but not in HepG2 cells (Figure 2f).

### 2.2. Effects of the Drugs on the Activities of SOD, GSH and the Level of MDA in Cells

**Figure 3:**
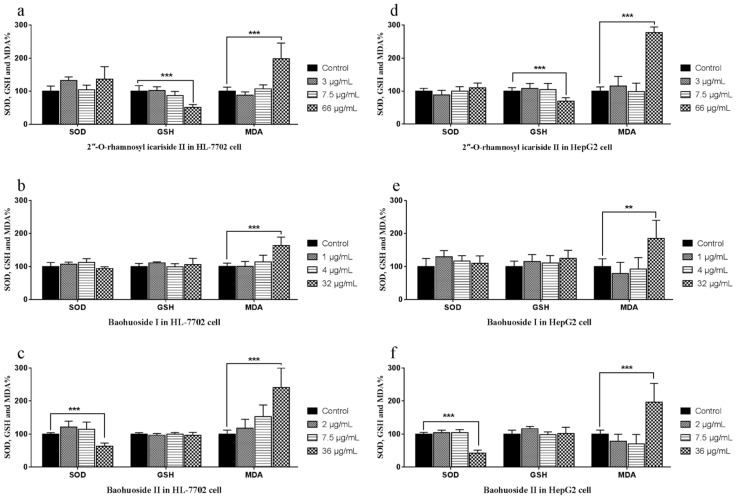
Effects of 2″-*O*-rhamnosyl icariside II, baohuoside I and baohuoside II on the SOD, GSH and MDA in HL-7702 and HepG2 cells at 24 h. Notes: The activities of SOD were significantly decreased by treating with high concentration of baohuoside II in HL-7702 (**c**) and HepG2 (**f**) cells. The activities of GSH were significantly decreased by treatment with high concentration of 2″-*O*-rhamnosyl icariside II in HL-7702 (**a**) and HepG2 (**d**) cells. The levels of MDA were significantly increased by treatment with high concentrations of all three drugs in HL-7702 (**a**–**c**) and HepG2 (**d**–**f**) cells. Each value is the mean ± SD of five experiments. * *p* < 0.05, ** *p* < 0.01, and *** *p* < 0.005 compared with the control group.

The SOD, GSH and MDA activity and level measurements are shown in Figure 3. The three drugs impact the monitoring indexes only at high concentrations. 2″-*O*-Rhamnosyl icariside II at 66 μg/mL significantly decreased GSH activity (*p* < 0.005) and significantly increased MDA level (*p* < 0.005) (Figure 3a,d) in both types of cells. Baohuoside I at 32 μg/mL caused the MDA level to increase significantly (*p* < 0.005) in both types of cells (Figure 3b,e). Baohuoside II at 36 μg/mL caused the SOD activity to decrease significantly (*p* < 0.005) and the MDA level to increase significantly (*p* < 0.005) (Figure 3c,f) in both types of cells.

### 2.3. Effects of Extracts and Compounds on the Generation of ROS

**Figure 4:**
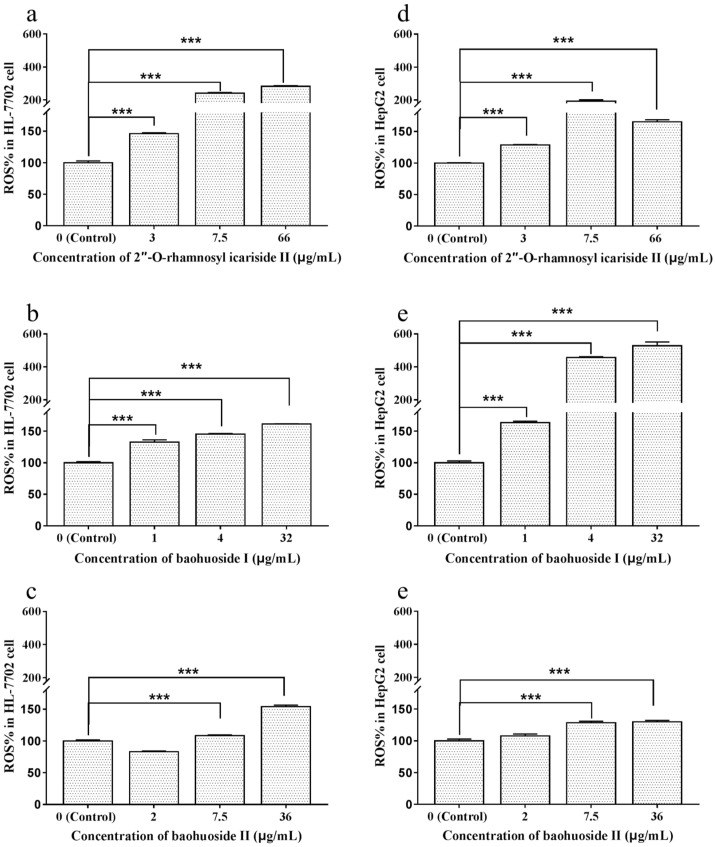
Effects of 2″-*O*-rhamnosyl icariside II, baohuoside I and baohuoside II on the ROS level in HL-7702 and HepG2 cells at 24 h. Notes: Except baohuoside II at low concentration, the other groups caused significant increases (*p* < 0.005) in HL-7702 (**a**–**c**) and HepG2 (**d**–**f**) cells. Each value is the mean ± SD of three experiments. * *p* < 0.05, ** *p* < 0.01, and *** *p* < 0.005 compared with the control group (ROS = reactive oxygen species).

**Figure 5:**
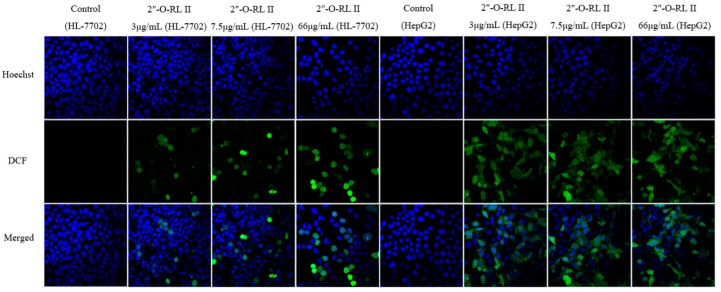

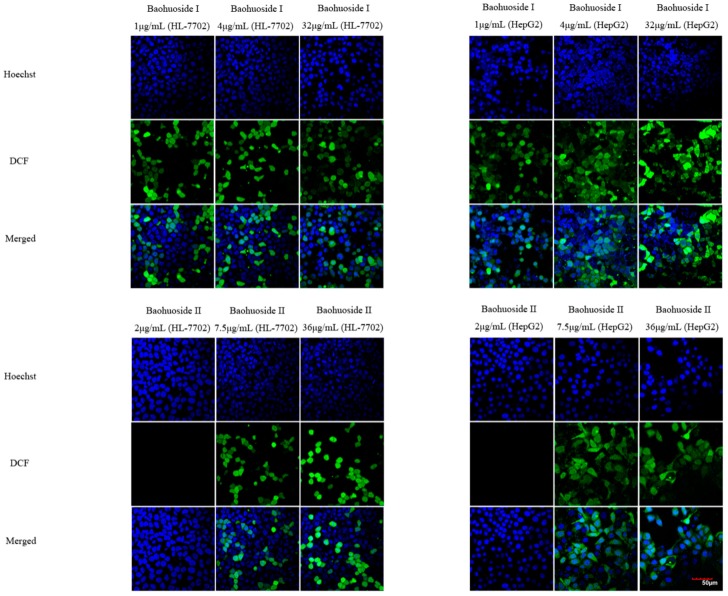
Effects of 2″-*O*-rhamnosyl icariside II, baohuoside I and baohuoside II on the fluorescence intensity of DCF in HL-7702 and HepG2 cells. *Notes*: Except baohuoside II at low concentration, the fluorescence intensity of DCF in the other groups was stronger than that of the control group. The scale bar corresponds to 50 μm. Magnification ×600 (2″-*O*-RL II = 2″-*O*-rhamnosyl icariside II.

To investigate the effects of the drugs on the generation of ROS in HL-7702 and HepG2 cells, fluorescence staining was performed, and the fluorescence intensity was determined by an FCM and observed by a confocal laser scanning microscope. The results calculated from the fluorescence intensity measurements are shown in Figure 4. After treatment with different concentrations of 2″-*O*-rhamnosyl icariside II (3, 7.5 and 66 μg/mL), baohuoside I (1, 4 and 32 μg/mL) and baohuoside II (7.5 and 36 μg/mL), the differences in the ROS level compared to the control groups were statistically significant (*p* < 0.005) in both types of cells. The images of ROS staining are shown in Figure 5. The DCF, which was generated by ROS in cells acting on DCFH-DA, was stained green, and the nucleus was stained blue. The confocal laser scanning microscopy data were consistent with the abovementioned FCM data. The fluorescence intensity of DCF in the groups of 2″-*O*-rhamnosyl icariside II and baohuoside I at each concentration and baohuoside II at middle and high concentrations were stronger than that of the control group.

### 2.4. Effects of Extracts and Compounds on the Change in MMP

**Figure 6:**
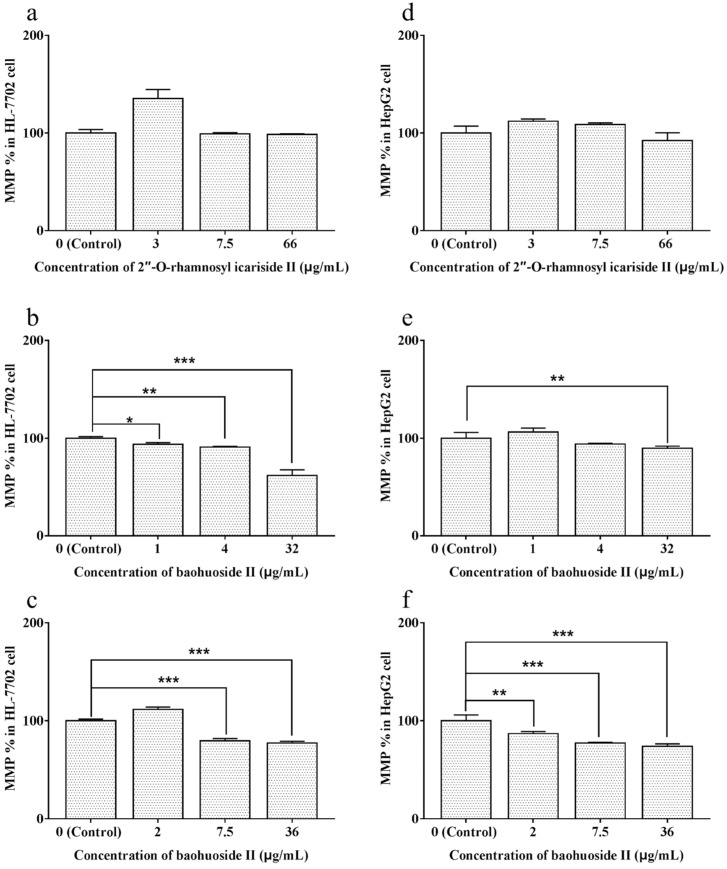
Effects of 2″-*O*-rhamnosyl icariside II, baohuoside I and baohuoside II on the MMP level in HL-7702 and HepG2 cells at 24 h. Notes: In HL-7702 cells, 2″-*O*-rhamnosyl icariside II (**a**) at each concentration did not cause a decrease in the MMP level, baohuoside I (**b**) at each concentration caused a significant decrease in the MMP level (*p* < 0.05, *p* < 0.01 and *p* < 0.005), and baohuoside II (**c**) at 7.5 and 36 μg/mL caused a significant decrease in the MMP level (*p* < 0.005). In HepG2 cells, 2″-*O*-rhamnosyl icariside II (d) at each concentration also did not cause a decrease in the MMP level, baohuoside I (**e**) at 32 μg/mL caused a significant decrease in the MMP level (*p* < 0.01), and baohuoside II (**f**) at each concentration caused a significant decrease in the MMP level (*p* < 0.01, *p* < 0.005 and *p* < 0.005). Each value is the mean ± SD of three experiments. * *p* < 0.05, ** *p* < 0.01, and *** *p* < 0.005 compared with the control group (MMP = mitochondrial membrane potential).

The results calculated from the fluorescence intensity values of MMP measured by FCM are shown in Figure 6. 2″-*O*-rhamnosyl icariside II at each concentration did not have an impact on MMP in both types of cells (Figure 6a,d). Baohuoside I at1 and 4 μg/mL caused significant decreases in the MMP level (*p* < 0.05, *p* < 0.01) in HL-7702 cell (Figure 6b), while at 32 μg/mL, a significant decrease (*p* < 0.01, *p* < 0.005) appeared in both types of cells (Figure 6b,e). Baohuoside II at 2 μg/mL caused a significant decrease in the MMP level (*p* < 0.01) in HepG2 cells (Figure 6f), while at 7.5 and 36 μg/mL, the significant decrease (*p* < 0.005) appeared in both types of cells (Figure 6c,f).

**Figure 7:**
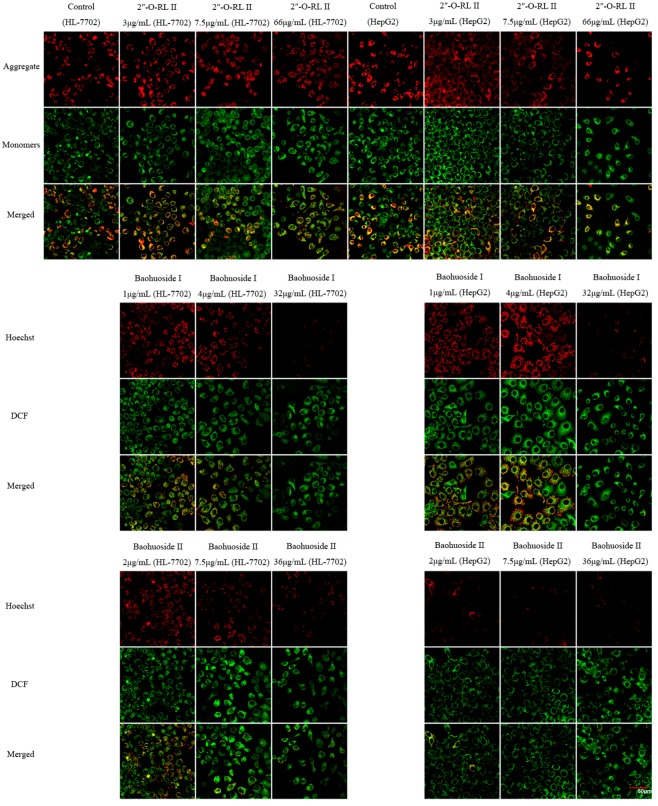
Effects of 2″-*O*-rhamnosyl icariside II, baohuoside I and baohuoside II on the fluorescence intensity of JC-1 in HL-7702 and HepG2 cells. Notes: 2″-*O*-rhamnosyl icariside II at each concentration did not cause fluorescence intensity changes. The fluorescence intensities of aggregates in the groups of baohuoside I and baohuoside II at different concentrations were weaker than that of the control group. The scale bar corresponds to 50 μm. Magnification ×600 (2″-*O*-RL II = 2″-*O*-rhamnosyl icariside II).

The images of MMP staining, determined by a confocal laser scanning microscope, are shown in Figure 7. The aggregates of JC-1 were stained red, and the monomers were stained green. In HL-7702 cells, after treatment with 2″-*O*-rhamnosyl icariside II, the staining results were similar to those of the control group. However, when HL-7702 cells were incubated with baohuoside I (1, 4 and 32 μg/mL) and baohuoside II (7.5 and 36 μg/mL), the red fluorescence intensity was weaker than that of the control group. In HepG2 cells, each group of 2″-*O*-rhamnosyl icariside II was also similar to the control group. However, the red fluorescence intensity of the group with baohuoside I (32 μg/mL) or baohuoside II (2, 7.5 and 36 μg/mL) was weaker. These data were consistent with the abovementioned FCM data.

## 3. Discussion

TCMs and natural herbs have a long history of use in clinical practice based on a unique traditional theory, especially in Asian countries [24]. As the global consumption of TCM products rapidly expanding and relevant research reports increasing, liver injury cases associated with TCM have attracted great concern in the last few years [25]. Therefore, TCM practitioners, researchers and patients should be aware of the hepatotoxic potential of TCM products.

Indeed, the cases of TCM-induced liver injury appear frequently with excessive, blinded, or inappropriate use [26,27]. In addition to individual factors involved in TCMs, the possible risk factors of TCMs’ hepatotoxicity are intrinsic to the TCM itself. On the one hand, TCMs have complex components, some of which may initiate liver injury after ingestion, and drug incompatibility and metabolic reactions can increase the risk of DILI [28,29]. On the other hand, the quality of TCMs may be influenced by hazardous materials (pesticides, heavy metals or mycotoxins), alternative plant species or unsafe processing, leading to increased hepatotoxicity [30,31,32,33]. Thus, it is difficult to conclusively identify the valid reason underlying TCM products’ hepatotoxicity. To identify the main factor in toxicity and improve research efficiency, composite analyses of toxic manifestations in vivo, potentially toxic composition selection and toxic evaluation in vitro can be helpful in early research. Therefore, with a toxicity assessment of the extracts and the aim of identifying hepatotoxic compounds, we conducted research on the toxicity of 2″-*O*-rhamnosyl icariside II, baohuoside I and baohuoside II in vitro.

Although the exact mechanism of DILI caused by TCMs remains largely unknown, it appears to involve two pathways: direct hepatotoxicity and adverse immune reactions [34,35]. Direct hepatotoxicity is often caused by the direct action of certain hepatotoxic compounds in TCMs, or reactive metabolites of compounds, against hepatocytes. In the process of producing toxicity, the hepatotoxic compounds or the reactive metabolites interact with cellular macromolecules such as lipids, nucleic acids and proteins, resulting in lipid peroxidation, DNA damage, protein dysfunction, enzymatic activity change and oxidative stress. Additionally, these toxic substances may induce disruption of ionic gradients and intracellular calcium stores, leading to mitochondrial dysfunction and loss of energy production. This impairment of cellular function can finally culminate in cell apoptosis or death and possible liver failure [4]. In response to this damaging situation, we chose a series of hepatotoxic indicators to comprehensively and effectively evaluate the toxicity of compounds. When liver cells suffer damage and direct toxicity by drugs, the cell membrane is destroyed, resulting in the leakage of ALT, AST and LDH into the extracellular environment; thus, detecting the activity of ALT, AST and LDH in the extracellular environment can illustrate the strength of direct hepatotoxicity of drugs. In addition, SOD, GSH and MDA are also important hepatotoxic indicators and are correlated with oxidative stress. ROS are indicators directly reflecting the intracellular level of oxidative stress, and the oxidative injury in liver cells caused by ROS can damage the structures and functions of the cytomembrane, mitochondrial membrane and DNA and regulate a variety of apoptotic signals and initiate the apoptosis process. A decrease in MMP is known to be an early event in apoptosis.

In this study, the results of the analysis point to the conclusion that three compounds showed significant dose-toxic effect relationships, and the results of each index in two cell models are similar. 2″-*O*-rhamnosyl icariside II at high concentration (66 μg/mL) cause increased extracellular AST and LDH activity, decreased intracellular GSH and increased MDA, and at each concentration, could cause increases in ROS. Thus, once 2″-*O*-rhamnosyl icariside II exerts toxic effects, it may directly damage liver cell structure and enhance oxidative stress. Baohuoside I and baohuoside II at high concentrations (32 μg/mL and 36 μg/mL) also influenced the level of AST, LDH and MDA, and at each concentration, ROS increased. Additionally, baohuoside II at high concentration (36 μg/mL) caused decreased SOD. Baohuoside I and baohuoside II at a certain concentrations could significantly decrease the MMP level. Therefore, the following can be inferred: firstly, the similarity of cytotoxicity effect between baohuoside I and baohuoside II must arise from the similar molecular structure involved in the structure-toxicity relationship; secondly; compared with 2″-*O*-rhamnosyl icariside II, due to the significant raising of ROS and dropping of MMP, the influence of these two compounds on toxicity seemed to induce apoptosis through oxidative stress and then achieve toxic effects on liver cells, which means their toxic mechanism may be associated with apoptotic pathways. Although the direct apoptosis detection was not carried out in this article, the thorough researches of apoptosis are underway.

After entering the body, the flavonoids of Herba Epimedii, through various pathways, will metabolize to secondary glycosides, which are beneficial to absorbing into blood and increasing the effects of themselves [23]. Baohuoside I not only exists in its amount in the extract, but also is one of the main metabolites of other compounds of the extract, so its amount will be increased in vivo by metabolisis, based on the dose-toxic effect relationship, which raises the risk of toxicity. From this we surmise that baohuoside I is more likely involved in the hepatotoxicity of Herba Epimedii than 2″-*O*-rhamnosyl icariside II and baohuoside II. Moreover, the hepatotoxicity of Herba Epimedii, like other TCMs, is probably due to the combined effects of multiple components, and the possibility exists of specific hepatotoxicity related to idiosyncratic and hypersensitive responses to drugs. These issues will be among our focus in future studies.

## 4. Materials and Methods

### 4.1. Chemicals and Reagents

HL-7702 and HepG2 cells were purchased from China Infrastructure of Cell Line Resources (Beijing, China). 2″-*O*-Rhamnosyl icariside II (PubChem CID: 5318987), baohuoside I (PubChem CID: 113558-15-9), and baohuoside II (PubChem CID: 55395-07-8) were purchased from Yuanye Bio-Technology Co. Ltd. (Shanghai, China). Dimethyl sulfoxide (DMSO) was from Sigma-Aldrich (St. Louis, MO, USA). Assay kits for total protein (bicinchoninic acid (BCA), number A045-4), superoxide dismutase (SOD, number A001-3), glutathione (GSH, number A006-2), and malondialdehyde (MDA, number A003-4) were purchased from NanJing JianCheng Bioengineering Institute (NanJing, China). Mitochondrial membrane potential assay kit with JC-1 (number C2006) for ROS and reactive oxygen species assay (number S0033) for MMP were obtained from Beyotime Institute of Biotechnology (Haimen, China).

### 4.2. Cell Culture

HL-7702 and HepG2 cells were cultured in Roswell Park Memorial Institute (RPMI) 1640 medium (GIBCO, Grand Island, NY, USA) supplemented with 10% heat-inactivated fetal bovine serum (PAN-Biotech GmbH, Aidenbach, Germany), 100 U/mL penicillin, and 100 μg/mL streptomycin. The cells were kept in a humidified atmosphere with 5% CO_2_ at 37 °C. When reaching 90% confluence, the cells were trypsinized using 0.25% trypsin with 0.02% EDTA and then passaged. HL-7702 cells and HepG2 cells were used at passages 22–39 and 18–35.

### 4.3. Activity Assessment of ALT, AST and LDH in Cells

The in vitro activities of alanine aminotransferase (ALT), aspartate aminotransferase (AST) and lactate dehydrogenase (LDH) in vitro were assayed as markers of hepatotoxicity. HL-7702 and HepG2 cells were prepared as single-cell suspensions at densities of 5 × 10^4^ cells/mL and 1 × 10^4^ cells/mL and then dispensed into 96-well plates (Corning, Corning, NY, USA) at a total volume of 100 μL per well. The standard compounds were separately dissolved in medium with 0.5% (*v*/*v*) DMSO and divided into three concentrations for in vitro studies based on 24 h IC_50_ values in HL-7702 and the contents in Herba Epimedii extract: low (equal to the content when the extract reach the maximum nontoxic concentration), middle (equal to the content when the extract reach the concentration of IC_50_ value) and high (equal to the concentration when the compound reach IC_50_ value). 2″-*O*-rhamnosyl icariside II was studied at 3, 7.5 and 66 μg/mL; baohuoside I was studied at 1, 4 and 32 μg/mL; baohuoside II was studied at 2, 7.5 and 36 μg/mL. After culturing for 24 h, the medium in each well was removed and replaced with fresh medium or medium containing different drugs at various concentrations. After incubation for an additional 24 h, the culture medium in each well was collected and ALT, AST and LDH activities were detected according to the pyruvic acid method [36] with a CX4 Pro automatic biochemical analyzer (Beckman, Brea, CA, USA). The activities were expressed as the percentages of the activities over the corresponding control groups.

### 4.4. Activity Assessment of SOD, GSH and MDA in Cells

The activities of SOD, GSH and MDA in both cell types were assayed as indices of hepatotoxicity. HL-7702 and HepG2 cells were prepared as single cell suspensions at densities of 5 × 10^4^ cells/mL and 1 × 10^4^ cells/mL and then dispensed into 12-well plates (Corning) at a total volume of 1.5 mL per well. After culturing for 24 h and then incubating with fresh medium or medium containing different drugs at various concentrations for an additional 24 h, the cells were collected and dispensed into 0.5 mL of an appropriate solution based on the requirements for the measurement of different indices. The cells were then disrupted by ultrasonication with an Ultrasonic Cell Cracker (Scientz Biotechnology Co., Ningbo, China). The activities of SOD, GSH and MDA in the cell suspension were measured and calculated according to the manufacturer’s instructions for the assay kits, and the activities were expressed as the percentages of the activities over the corresponding control groups.

### 4.5. Measurement of Intracellular ROS Levels

Intracellular ROS production was assessed with a flow cytometer (FCM; Becton Dickinson, Franklin Lakes, NJ, USA) and visualized on a confocal laser scanning microscope (Olympus, Tokyo, Japan) using an ROS assay kit. In brief, cells were seeded in 12-well plates or 35-mm glass-bottom culture dishes (MatTek Corporation, Ashland, MA, USA) and treated with different drugs in a manner similar to the method used for activity assessment of SOD, GSH and MDA. Thereafter, the cells were incubated with 6-carboxy-2′,7′-dichlorodihydrofluorescein diacetate (DCFH-DA) at a final concentration of 10 μM for 30 min at 37 °C in the dark. After the cells were harvested, the relative fluorescence levels were quantified by an FCM (excitation at 485 nm and emission at 535 nm) according to the manufacturer’s instructions. The cells in the glass-bottom culture dishes were washed three times with PBS, and the cell nuclei were stained with Hoechst 33342 (Solarbio, Shanghai, China) for 10 min at 37 °C in the dark. After washing again, the morphology and fluorescence of the ROS were observed with a confocal laser scanning microscope and the images were processed using the analysis software. The ROS levels were expressed as the percentages of the ROS levels over the corresponding control groups.

### 4.6. Assessment of MMP

Changes in MMP were also evaluated by an FCM and a confocal laser scanning microscope using an MMP assay kit. The two types of cells were seeded and treated in a manner similar to the method used for the measurement of intracellular ROS levels. Thereafter, the cells were incubated with working solutions of the cationic fluorochrome JC-1 for 30 min at 37 °C in the dark. JC-1 accumulates in the mitochondria as red fluorescent aggregates at an intact MMP but exists as a green fluorescent monomeric form at decreased MMPs. The fluorescence intensities detected in the fluorescence channels FL-1 (green) and FL-2 (red) were analyzed by an FCM, and the fluorescence images were collected by a confocal laser scanning microscope. The changes in MMP were expressed as the percentages of the changes in MMP over the corresponding control groups.

### 4.7. Statistical Analysis

Each set of results shown is representative of three separate experiments. Statistical analyses were performed with the SPSS software suite, version 17.0 (SPSS, Inc., Chicago, IL, USA). All data are expressed as the mean ± standard deviation (SD). Statistical analysis was performed using one-way ANOVA followed by the Bonferroni test for individual comparisons between the means of multiple groups and corresponding control groups. Significance was set at *p* < 0.05.

## 5. Conclusions

This article aimed to evaluate the influence of hepatotoxic suspicions of Herba Epimedii, 2″-*O*-rhamnosyl icariside II, baohuoside I and baohuoside II on liver cells in vitro. Through the determination of a series of cytotoxicity indices, including ALT, AST, LDH, SOD, GSH, MDA, ROS and MMP, the toxic manifestations of three compounds at certain concentrations were revealed and exhibited dosage reliability. Among the tested substances, baohuoside I was shown to be more closely correlated with the liver injury induced by Herba Epimedii, and its toxicity mechanism(s) may be involved in increasing oxidative stress and induction of apoptosis. Considering the increasing use of TCMs, including Herba Epimedii, these findings may serve as an impetus to monitor TCM safety and to further explore the relevant knowledge to avoid TCM-induced liver injury.
